# Clinical Features and Neurosurgical Management of Metastatic Intradural Extramedullary Renal Cell Carcinoma

**DOI:** 10.7759/cureus.33618

**Published:** 2023-01-10

**Authors:** Omron Hassan, Kelly Gassie, Anshit Goyal, Stephanie Foskey, Kingsley Abode-Iyamah

**Affiliations:** 1 Clinical Sciences, Touro University Nevada College of Osteopathic Medicine, Henderson, USA; 2 Neurological Surgery, Mayo Clinic, Jacksonville, USA; 3 Neurological Surgery, Mayo Clinic, Rochester, USA

**Keywords:** back pain, intradural extramedullary spine tumors, spinal cord, myelopathy, metastases, renal cell carcinoma

## Abstract

Intradural extramedullary metastasis of renal cell carcinoma is exceedingly uncommon, and only 19 cases have been reported in the literature. It is thought to metastasize from the kidneys through venous networks or along nerves and may also spread from brain metastases through cerebrospinal fluid. We present a 52-year-old female, two years after a nephrectomy with myelopathic symptoms, who was found to have thoracic intradural extramedullary metastasis from renal cell carcinoma. The thoracic tumor was resected without any added deficit, but an additional brain mass was found on postoperative imaging. The present case and a literature review were discussed to explore considerations for neurosurgical intervention in similar patients, evaluate surgical outcomes, and highlight current theories on routes of metastasis. Given the risk of neurological decline in patients with metastatic intradural renal cell carcinoma, surgical resection should be considered upon its discovery, and postoperative surveillance imaging is encouraged.

## Introduction

Renal cell carcinoma (RCC) is an adenomatous neoplasm that arises from the renal tubular epithelium. Because it often silently metastasizes, it may be discovered through clinical manifestations of other compromised bodily systems. RCC has been reported to metastasize in 25% of patients prior to detection, but intradural involvement is rarely reported in the literature [1]. Spinal tumors have been reported within the thecal sac only up to 6% of the time, and intradural extramedullary metastases have been reported in 0.9-2.1% of autopsied cancer patients [1-4]. Intradural metastases are most commonly found in the cervical spine, followed by the thoracic and lumbar spine, and RCC makes up 4% of spinal metastasis [1]. Patients with intradural tumors of the spine may present with progressive myelopathic symptoms or other clinical signs suggestive of a herniated disc. High-resolution MRI is the diagnostic tool of choice for identifying intradural tumors, and RCC may appear well-defined and hypointense on T1-weighted, hyperintense on T1-weighted post-contrast, and hypointense on T2-weighted images [1,5]. Differential diagnosis may include more commonly encountered spinal tumors. However, prior diagnosis of RCC should lead to a higher level of suspicion of intradural metastasis, even though intradural tumors originating from non-primary malignancies are uncommon [6]. Surgical resection is suggested for intradural extramedullary renal cell carcinoma (IERC) for the best long-term outcome. This can be done through a laminectomy followed by an exploration of the subdural space. Adequate preparation should be made when approaching these tumors because of the risk of durotomy and injury to neural structures. Furthermore, adenomatous lesions may bleed excessively, making planes between the tumor and the spinal cord challenging to identify. We report a case of a female patient who presented with myelopathy. Upon imaging, she was found to have an intradural extramedullary spinal tumor confirmed to be metastatic clear cell RCC. We aim to discuss this patient's treatment course, including relevant clinical and imaging findings and further discuss neurosurgical treatment and theories for the routes of metastasis for IERC through a review of the literature.

## Case presentation

**Figure 1:**
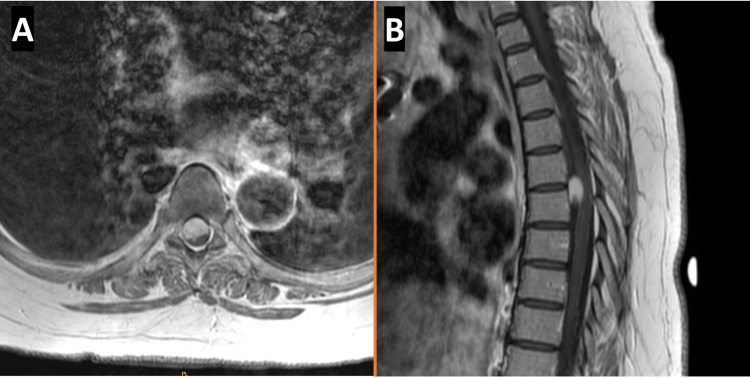
Preoperative T1-weighted post-contrast MRI with axial (A) and sagittal (B) views showing the IERC. IERC: Intradural extramedullary renal cell carcinoma.

A 52-year-old female patient with a history of RCC, who previously underwent a right-sided nephrectomy two years ago, followed by chemotherapy and radiation, presented to the ED with gait disturbances. She was recently seen at another center where a neurosurgeon attempted surgical resection of a thoracic spinal tumor but aborted their surgery due to loss of intraoperative neuromonitoring. Two months later, she had abrupt lower extremity weakness, sensory deficit, and gait instability. She subsequently came to our center ambulating with a walker. Upon physical examination, she was found to have bilateral 5/5 strength in knee flexion/extension and foot dorsiflexion/plantarflexion. Her lower extremity reflexes were 3+ bilaterally, and she had a positive right Babinski sign. An MRI of her thoracic spine revealed a 1.7 cm craniocaudally, 0.9 cm anteroposterior, and 1.1 cm coronal intradural extramedullary tumor between T5-6 that was hyperintense and homogenous on a T1-weighted post-contrast image (Figure 1). Given the patient's myelopathic symptoms, imaging findings, and history of RCC, surgical resection of the tumor was determined to be the best course of treatment to relieve her symptoms and improve her long-term outcome.

Operative course 

The patient was taken to the operating room and placed prone in head pins, and a midline incision was made between T5 and T6 along the prior incision site. A laminectomy was performed, and intraoperative ultrasound was used to identify and localize the tumor prior to durotomy. Due to the tumor's location, a dentate ligament on the right side was freed, and the traversing right T4 nerve root was transected, allowing the resection of the tumor in a piecemeal fashion. There were no intraoperative complications. A specimen was sent for frozen sectioning, and the results found the tumor to be consistent with a metastatic clear cell RCC, which was the same as the primary renal tumor.

Outcome and follow-up

**Figure 2:**
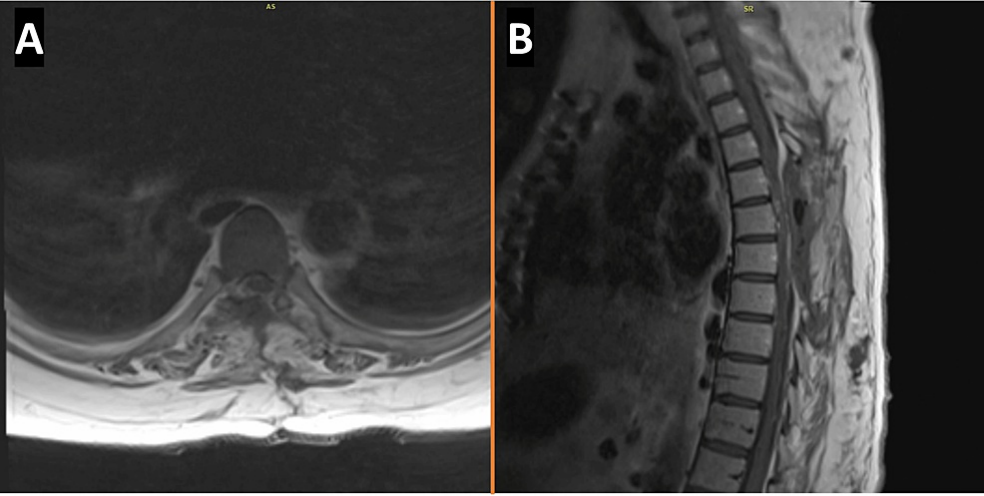
Postoperative T1-weighted post-contrast MRI in axial (A) and sagittal (B) views without residual tumor.

Immediate recovery was uncomplicated, and the patient's presenting symptoms resolved within the first postoperative day. A physical examination revealed bilateral lower extremity strength of 5/5 in knee flexion/extension and foot plantarflexion/dorsiflexion. She no longer had a positive Babinski sign, and her reflexes were 2+ bilaterally, but she had a residual sensory deficit. A postoperative MRI demonstrated complete resection of the tumor, and the thecal sac was no longer compressed (Figure 2). On postoperative day 5, the patient reported subjective mental status changes, and an MRI of the brain revealed a small, right-sided cerebellar mass suspected to be a metastatic tumor. However, no plans for immediate operation were made at the time. At a one-month follow-up visit, she had no new neurologic deficits, and she reported further improvement in her sensory deficit. Plans for follow-up care regarding her brain metastasis and monitoring for spinal tumor recurrence were made.

## Discussion

A review of the published English-language literature on the PubMed database from 1991 to the present (October 2021) was performed for all prior cases of IERC, reported to be surgically removed. Articles reporting intradural intramedullary RCC metastases, non-intradural metastases of the spinal column, or other types of spine tumors were excluded. The search resulted in 18 articles that were published between 1999 and 2021 with a total number of 19 patients ranging in age from 36 to 84 (13 male, six female) with an average age of 61.5 (Table 1) [1-18]. MRI was the imaging modality of choice for preoperative tumor diagnosis in all studies. Other concomitant metastases reported were to the lungs (25%), brain (18.8%), mediastinum (6.3%), tibia (6.3%), vertebrae (6.3%), and liver (6.3%). The reported complications throughout the studies were postoperative weakness and sensory deficit, but there were no reports of intraoperative complications. The most commonly reported area of the spine with RCC metastasis was the lumbar spine, followed by the thoracic and cervical spine. Pathologic subtypes of RCC, when reported, were clear cell (69.2%), papillary (23.1%), and small cell carcinoma (7.7%). There were two reported cases where the initial diagnosis of RCC was through patients presenting with symptoms from their spinal tumor [4,14].

**Table 1:** Operative cases of intradural extramedullary renal cell carcinoma.

Article	Age/Sex	Clinical Presentation	Size (cm)	Spinal Levels	Additional Metastasis	Prior Treatment	Subtype	Adverse Events
Maxwell et al. 1999 [3]	84/M	Back Pain Radiculopathy	2.5x1.5	L2-3	-	-	Clear Cell	-
Mak et al. 2001 [6]	59/M	Back Pain Paresthesia Incontinence	-	L2	Vertebrae	Nephrectomy Radiotherapy	-	-
Takada et al. 2003 [1]	61/M	Back Pain Radiculopathy Paresthesia Incontinence	2.5x1.5	L3	Lung	Nephrectomy Lung Resection Interferon Therapy	Papillary	Foot Weakness
Kubota et al. (2004) [2]	68/M	Back Pain Radiculopathy	2.5x1.3	L3	Lung	Nephrectomy Pneumonectomy	-	-
Gaetani et al. (2004) [7]	36/F	Cauda Equina Syndrome	3x1.5	L3-4	Brain	Nephrectomy Radiotherapy	Clear Cell	-
Alfieri et al. (2005) [8]	67/F	Back Pain Incontinence Weakness	6.2x1.6	L3-5	-	Nephrectomy	Papillary	Limited Neurological Improvement
Kim et al. (2009) [9]	41/M	Back Pain Radiculopathy	1.5x1.1	L2	Lung	Nephrectomy Chemotherapy	Clear Cell	-
Dobson et al. (2013) [4]	81/F	Cauda Equina Syndrome	2.7x1.5x1.5	L2	-	-	Clear Cell	Permanent Neurologic Deficit
Jost et al. (2009) [10]	82/M	Hemiparesis Neck Pain	-	C6-7	Brain	Nephrectomy Radiotherapy	Clear Cell	-
Lin et al. (2011) [11]	68/M	Back Pain Paraparesis Urinary Retention	1.5x1.5x2	T12-L1	-	Nephrectomy	Clear Cell	Lower Extremity Weakness
Ji et al. (2013) [12]	68/M	Back Pain Radiculopathy Shuffling Gait	3.2x1.3cm	T12-L1	Tibia	Nephrectomy	Clear Cell	-
Strong et al. (2013) [13]	49/F	Radiculopathy Weakness	2x2.7x1.6	L4	-	Nephrectomy	-	-
Strong et al. 2013 [13]	72/M	Hyperreflexia	1.4x1.1	L2	-	-	-	Residual Numbness
Srinivasan et al. (2014) [14]	40/M	Paresthesia Incontinence	-	L4-S2	-	-	Small Cell	-
Capek et al. (2016) [15]	61/F	Back Pain Radiculopathy	-	T12	-	Nephrectomy	Clear Cell	-
Heary et al. (2014) [16]	54/M	Back Pain Paresthesias	-	T2-4	-	-	-	-
Ganapathy et al. (2018) [17]	54/M	Radiculopathy	3.2x1.9x1.4	L3-4	-	Nephrectomy Chemotherapy Radiotherapy	-	-
Carminucci et al. (2020) [5]	68/M	Radiculopathy	1.5	C3-4	Liver Lungs Mediastinum Brain	Nephrectomy Chemotherapy Radiotherapy	Papillary	-
Ali et al. (2021)	55/F	Back Pain Paresthesias	2.5x1.4	L3-4	-	Nephrectomy Chemotherapy Radiotherapy	Clear cell	-

Clinical presentation of intradural spinal tumors often includes radiculopathy, myelopathy, or cauda equina syndrome. The extent of tumor progression in which patients become symptomatic may be suggested by size based on the literature findings, as IERC of at least 1.3 cm in any dimension may lead to symptoms in patients. There was disparity throughout the literature in the reporting of tumor size, and there was a small sample size which prevents further determination of the threshold for clinically symptomatic IERC based on size. RCC forms through a malignant transformation of renal epithelial cells, and given its high rate of metastasis, there are multiple theories on potential pathways for its metastatic course. Venous drainage communicating with the vertebral venous plexuses may be a route for the spread of RCC from the kidneys due to small anastomotic complexes [2,10]. This may be possible because blood flow within the vertebral venous system can be bidirectional due to its valveless system, which allows metastatic seeding that has been demonstrated on angiograms [10]. Takada T et al. suggest that about 90% of metastatic spinal tumors occur alongside brain metastases, which may be a result of cerebrospinal fluid communication within the thecal sac [1]. In our present case, our patient had brain and spine tumors, and our literature review found that 18.8% of IERC patients also have brain metastases. Because of the higher likelihood of co-occurrence upon discovery of brain or spinal RCC metastasis, further imaging in these patients for detection of metastasis may allow lesions to be discovered prior to becoming large and symptomatic. Resection may be easier, which, as a result, may lead to fewer postoperative neurological deficits, particularly in the lower spine, where nerves were commonly injured or sacrificed throughout studies [1,4,8,13].

When patients who present with clinical signs of the spinal cord or nerve root compression are encountered, the differential diagnosis should include tumors alongside suspicion of herniated discs, spinal stenosis, and fractures. However, in patients with prior malignancy, the suspicion of a metastatic tumor should be even greater. Intradural RCC metastases, on average present 5.8 years after the discovery of a primary tumor, and they rarely are the initial symptomatic lesions that lead to the detection of RCC [4,14-15]. Metastatic tumors have been found to grow quicker compared to primary tumors [2]. Therefore, in patients followed longitudinally for small spine tumors without a prior cancer diagnosis, a fast growth rate suggests a metastatic lesion. Further imaging may be performed to screen for a potential primary site of malignancy [2].

In patients with a history of RCC who present with spinal lesions that may be metastatic tumors, surgical resection is suggested over radiation therapy alone, as surgical resection has been found to increase the overall length of survival in patients [7]. Gaetani P et al. suggested surgical resection of suspected intradural RCC metastasis immediately after their discovery, regardless of the patient's symptom severity [7]. Despite the benefit of surgical resection, adjuvant radiotherapy may further reduce intradural recurrence despite RCC's tendency to be relatively resistant to radiotherapy [4,10]. Dobson GM et al. found that 93% of patients with intradural RCC spinal metastasis die within 15 months of their diagnosis. However, refinement of the timing of surgery, radiotherapy, and chemotherapy may prolong survival [4].

Despite the reduction in recurrence that comes with resection of IERC, intradural surgery comes with the risks of nerve injury. Patients should be given clear expectations regarding the likelihood of having residual or even new neurologic deficits after their surgery. Several cases in our review discussed nerve injury due to the sacrifice of nerve roots that were encased by tumors, and 31.3% of studies reported adverse events related to neurologic injury [1,8,11,13].

## Conclusions

Despite patients with RCC undergoing nephrectomy and adjuvant therapy, spinal metastases may occur. Early detection followed by immediate surgical resection may increase the length of survival and reduce discomfort in patients while their metastatic disease progresses. Furthermore, surveillance imaging for metastasis in patients with RCC and consideration for metastatic disease when encountering a spinal tumor without a history of other malignancy may further improve the management of IERC.

## References

[1] Takada T, Doita M, Nishida K, Miura J, Yoshiya S, Kurosaka M (2003). Unusual metastasis to the cauda equina from renal cell carcinoma. Spine.

[2] Kubota M, Saeki N, Yamaura A, Iuchi T, Ohga M, Osato K (2004). A rare case of metastatic renal cell carcinoma resembling a nerve sheath tumor of the cauda equina. J Clin Neurosci.

[3] Maxwell M, Borges LF, Zervas NT (1999). Renal cell carcinoma: a rare source of cauda equina metastasis. Case report. J Neurosurg.

[4] Dobson GM, Polvikoski T, Nissen JJ, Holliman D (2013). Cauda equina syndrome secondary to intradural renal cell carcinoma metastasis haemorrhage. Br J Neurosurg.

[5] Carminucci A, Hanft S (2020). Intradural extramedullary spinal metastasis of renal cell carcinoma: illustrative case report and comprehensive review of the literature. Eur Spine J.

[6] Mak KH, Kwok JC (2001). Intradural spinal metastasis from renal cell carcinoma: a case report. J Orthop Surg (Hong Kong).

[7] Gaetani P, Di Ieva A, Colombo P, Tancioni F, Aimar E, Debernardi A, Rodriguez Y Baena R (2004). Intradural spinal metastasis of renal clear cell carcinoma causing cauda equina syndrome. Acta Neurochir (Wien).

[8] Alfieri A, Mazzoleni G, Schwarz A, Campello M, Broger M, Vitale M, Vigl EE (2005). Renal cell carcinoma and intradural spinal metastasis with cauda equina infiltration: case report--part II. Spine (Phila Pa 1976).

[9] Kim DY, Lee JK, Moon SJ, Kim SC, Kim CS (2009). Intradural spinal metastasis to the cauda equina in renal cell carcinoma: a case report and review of the literature. Spine (Phila Pa 1976).

[10] Jost G, Zimmerer S, Frank S, Cordier D, Merlo A (2009). Intradural spinal metastasis of renal cell cancer. Report of a case and review of 26 published cases. Acta Neurochir (Wien).

[11] Lin TK, Chen SM, Jung SM (2011). Solitary intradural extramedullary metastasis of renal cell carcinoma to the conus medullaris. Kaohsiung J Med Sci.

[12] Ji GY, Oh CH, Kim SH, Shin DA, Kim KN (2013). Intradural cauda equina metastasis of renal cell carcinoma: a case report with literature review of 10 cases. Spine (Phila Pa 1976).

[13] Strong C, Yanamadala V, Khanna A, Walcott BP, Nahed BV, Borges LF, Coumans JV (2013). Surgical treatment options and management strategies of metastatic renal cell carcinoma to the lumbar spinal nerve roots. J Clin Neurosci.

[14] Kumar S, Srivastava T, Tejwani S (2014). A rare association of intracranial vertebral artery fenestration with nonaneurysmal perimesencephalic subarachnoid hemorrhage. Neurol India.

[15] Capek S, Krauss WE, Amrami KK, Parisi JE, Spinner RJ (2016). Perineural spread of renal cell carcinoma: a case illustration with a proposed anatomic mechanism and a review of the literature. World Neurosurg.

[16] Heary RF, Agarwal N, Barrese JC, Barry MT, Baisre A (2014). Metastatic renal cell carcinoma, with a radiographically occult primary tumor, presenting in the operative site of a thoracic meningioma: long-term follow-up: case report. J Neurosurg Spine.

[17] Ganapathy S, Gopal S, Godhani N, Raju A (2018). Isolated cauda equina metastasis from renal cell carcinoma - A rare cause of intradural-extramedullary compression. Indian J Neurosci.

[18] Ali S, Qasim A, Salah R, Sarwar MR, Usman M, Shams S (2021). Isolated late intradural cauda equina metastasis of renal cell carcinoma. Surg Neurol Int.

